# Links between Autism Spectrum Disorder Diagnostic Status and Family Quality of Life

**DOI:** 10.3390/children4040023

**Published:** 2017-04-03

**Authors:** Andrew G. McKechanie, Vivien J. Moffat, Eve C. Johnstone, Sue Fletcher-Watson

**Affiliations:** 1 Clinical Research Fellow and Honorary Consultant Psychiatrist, The Patrick Wild Centre, The University of Edinburgh, Edinburgh EH10 5HF, UK; 2 The Salvesen Mindroom Centre, The University of Edinburgh, Edinburgh EH9 1UW, UK; 3 Division of Psychiatry, The University of Edinburgh, Edinburgh EH10 5HF, UK; vivien.moffat@btinternet.com; 4 Professor Emeritus of Psychiatry and Honorary Assistant Principal, Mental Health Research Development and Public Understanding of Medicine, Division of Psychiatry, The University of Edinburgh, Edinburgh EH10 5HF, UK; E.Johnstone@ed.ac.uk; 5 Chancellor’s Fellow, The Patrick Wild Centre, The University of Edinburgh, Edinburgh EH10 5HF, UK; Sue.Fletcher-Watson@ed.ac.uk

**Keywords:** autism spectrum disorder, diagnosis, quality of life, stress

## Abstract

Quality of life is often relatively lowered in families of children with additional needs, and this may be particularly the case where additional needs are accompanied by an autism spectrum disorder (ASD). Here we explore the effects of diagnostic status specifically, comparing families with children with an ASD diagnosis with others who a) have additional needs but no signs of ASD; and b) have additional needs and signs of ASD but no diagnosis. Mothers (*n* = 76) of children with additional needs completed standardised questionnaires about quality of life, stress, service provision, child behaviour and presence and severity of ASD traits. In addition, a group of mothers of typically developing young people (*n* = 17) completed standardised questionnaires on individual and family quality of life and on the behaviour of their son or daughter. Mothers of typically developing young people had significantly higher individual and family quality of life scores than each of the three other groups. Increased severity of ASD was associated with increased maternal stress, which in turn was associated with decreased family and maternal quality of life. The group reporting the lowest quality of life and the highest stress were the mothers of individuals with signs of ASD but no diagnosis. This pattern did not seem to be explained by lack of access to services, or rates of intellectual disability or challenging behaviour in this sub-group. The finding that poor quality of life and high stress was most apparent in the sub-group of mothers with children who had signs of ASD but did not have a diagnosis of ASD suggests that an interesting topic for further investigation is whether receipt of a diagnosis itself can positively influence quality of life and levels of maternal stress.

## 1. Introduction

Autism spectrum disorder (ASD) refers to a constellation of conditions united by difficulties with typical social interaction and communication abilities, and the presence of restricted and repetitive behaviours as well as sensory hypo- and hypersensitivity [1]. ASD is also strongly associated with intellectual disability [2] and/or other mental health comorbidities (e.g.; anxiety, depression, obsessive-compulsive disorder (OCD), attention-deficit/hyperactivity disorder (ADHD)) [3] and can often give rise to behaviours which challenge family members and practitioners [4,5]. As a result, quality of life is often low, and stress levels are high in mothers of children and young people with ASD [6,7], even compared with other neurodevelopmental disorder categories [8,9]. Consistently lower maternal mental health has also been reported in longitudinal research [10]. Child characteristics such as challenging behaviour or intellectual disability have been reported as major stressors [11,12] while the perceived level of support has potential mediating effects [13]. 

In Scotland, there are increasing numbers of people being diagnosed with ASD, although this number is far short of the expected prevalence, suggesting that there may be substantial numbers of people with undiagnosed ASD [14]. We also know that waiting times for diagnosis after first concerns are raised can be long [15,16] and that parents report specific stress and dissatisfaction related to a delay in getting a definitive diagnosis of ASD [17,18,19]. Reasons for stress under these circumstances may include lack of access to appropriate support services, or systematic differences between children and young people who receive and do not receive an ASD diagnosis. In addition, lack of a definitive explanation for the difficulties being experienced by the child or young person, and their family, may be a stressor. 

This study aimed to investigate the quality of life status of families of children with possible undiagnosed ASD and unmet support needs. Identifying such a sample is a challenge, however we were fortunate to be able to conduct this study with a non-clinical sample derived from the education system and therefore free from some of the biases introduced by recruiting from within health services. We aimed to explore perceptions of mothers of young people with additional support needs in relation to stress, service provision and quality of life. A particular focus was the effect of having a family member with additional needs and ASD, and also the situation where the young person with additional needs may have an ASD that has not been recognised or diagnosed. In particular, the following research questions were addressed:

- How does the presence of ASD diagnosis impact upon family quality of life and stress?
- How is the relationship between diagnostic status and quality of life/stress mediated by: severity of ASD; presence of challenging behaviours; access to services?
- What are the characteristics of a young person with signs of ASD that is not diagnosed?

## 2. Results

Following screening with the Social Communication Questionnaire (SCQ) [20] and parental report of ASD diagnostic status, the children of these mothers were categorised as falling into one of four groups:

1. Having additional needs, a negative SCQ screening score and no diagnosis of ASD (*n* = 41).
2. Having additional needs, a positive SCQ screening score and an existing clinical diagnosis of ASD (*n* = 18).
3. Having additional needs, a positive SCQ screening score but no existing clinical diagnosis of ASD (*n* = 17).
4. Typically developing (TD), age-matched controls (*n* = 17).

Intelligence quotient (IQ) scores, measured with the Wechsler Adult Intelligence Scale [21] or the Wechsler Intelligence Scale for Children [22] as appropriate, were available for 82 of the 93 young people. Demographic data for each group is detailed in Table 1.

### 2.1. Impact of ASD Diagnosis on Maternal Quality of Life and Stress

**Figure 1:**
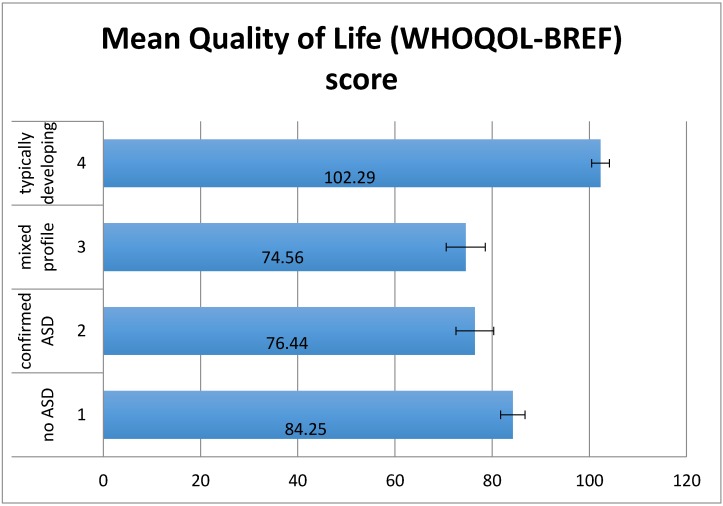
Mean World Health Organization Quality of Life-Brief Scale (WHOQOL-BREF) scores by group. Error bars show ±1 standard error. Mixed profile refers to group with additional needs, a positive SCQ screening score but no existing clinical diagnosis of ASD.

There was a significant effect of group on World Health Organization Quality of Life-Brief Scale (WHOQOL-BREF) scores (*F*(3,87) = 11.91, *p* < 0.01) (Figure 1). The hypothesised pattern of group scores was confirmed using planned contrasts for linear trend, *p* < 0.01. Mothers’ individual quality of life was significantly higher for the typically developing (TD) group than the other three groups (*t*(87) = −5.82, *p* < 0.01), Group 1 (low SCQ) had significantly higher scores than groups 2 and 3 (*t*(87) = −2.4, *p* < 0.05), but there was no significant difference between groups 2 and 3 (*t*(87) = −0.36, *p* = 0.72). Analysis of sub-domains of the WHOQOL-BREF showed that the TD group had significantly higher scores than the other three groups in each of the four domains but that there were no significant differences between the other three groups in any domain.

**Figure 2:**
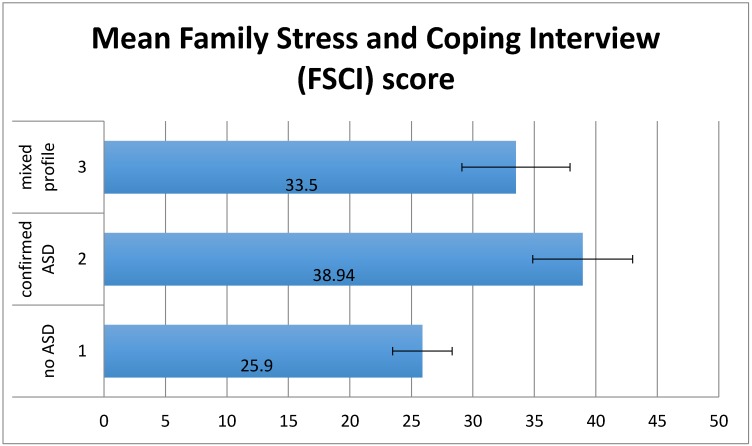
Mean FSCI scores by group. Error bars show ±1 standard error.

There was a significant effect of group on Family Stress and Coping Interview (FSCI) scores (*F*(2,70) = 4.12, *p* < 0.05) (Figure 2). No significant linear trend in the hypothesised direction was detected. Planned contrasts showed that whereas maternal stress was significantly lower for group 1 than group 2 and 3 (*t*(70) = 2.74, *p* < 0.01), there was no significant difference between group 2 and group 3 (*t*(70) = −0.96, *p* = 0.34).

### 2.2. Impact of ASD Diagnosis on Family Quality of Life

**Figure 3:**
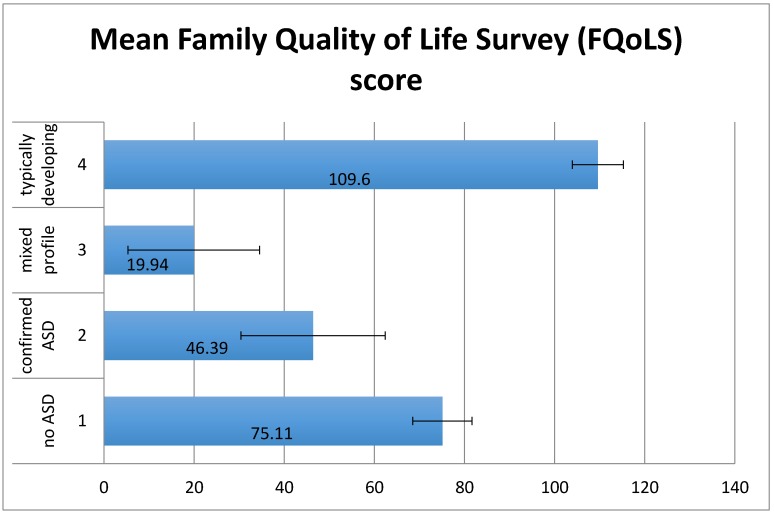
Mean FQoLS scores by group. Error bars show ±1 standard error.

There was a significant effect of group on Family Quality of Life Survey (FQoLS) scores (mothers’ mean score of satisfaction weighted by importance) (*F*(3,83) = 9.88, *p* < 0.01) (Figure 3). Planned contrasts for linear trend (*p* < 0.01), indicated that family quality of life (QoL) decreased proportionately, with the TD group having the highest QoL followed by group 1 then group 2 then group 3. Family QoL was significantly higher for the TD group than the other three groups (*t*(83) = −4.38, *p* < 0.01). In addition, family QoL was significantly higher for group 1 than for groups 2 and 3 (*t*(83) = −3.44, *p* < 0.01) and there was no significant difference between groups 2 and 3 (*t*(83) = −1.56, *p* = 0.12). 

The mothers of young people in groups 1–3 only completed the section of the FQoLS relating to disability issues. Results showed that group 1 scored significantly higher than groups 2 and 3 (*p* < 0.01) and groups 2 and 3 were not significantly different from each other.

### 2.3. Impact of ASD Features, Challenging Behaviour and Service Access

The control group was excluded from this analysis as their significantly lower scores for level of ASD and challenging behaviour and significantly higher scores for QoL would make it more difficult to detect relations amongst the other three groups. 

There were significant positive correlations between maternal stress and QoL and child level of challenging behaviour and SCQ scores (see Table 2). Possible confounding variables (age and IQ) showed no significant relationships with any other variable. Gender was not examined due to the preponderance of males especially in Groups 2 and 3 (with high SCQ scores)

We employed partial correlations to investigate whether levels of challenging behaviour could be affecting the group differences (Table 3). Relations between SCQ score and maternal stress and also between SCQ score and family quality of life were examined while controlling for challenging behaviour as measured by the Childhood Behaviour Checklist (CBCL) score. Results showed that there was still a significant positive correlation between SCQ score and maternal stress and a significant negative correlation between SCQ score and family quality of life when level of challenging behaviour was controlled. This suggests that while levels of challenging behaviour are having an effect on maternal stress and on family quality of life, they do not fully account for group differences in this study.

Correlations between self-reported service contact and usefulness with maternal and child ratings were carried out using Spearman’s correlation coefficient. Service contact and service usefulness were correlated with each other (*p* < 0.01). In addition, maternal stress (FSCI score) showed a positive correlation with service contact (*p* < 0.05). There were no other significant relations. 

### 2.4. Characteristics of Participants with and without an ASD Diagnosis

**Figure 4:**
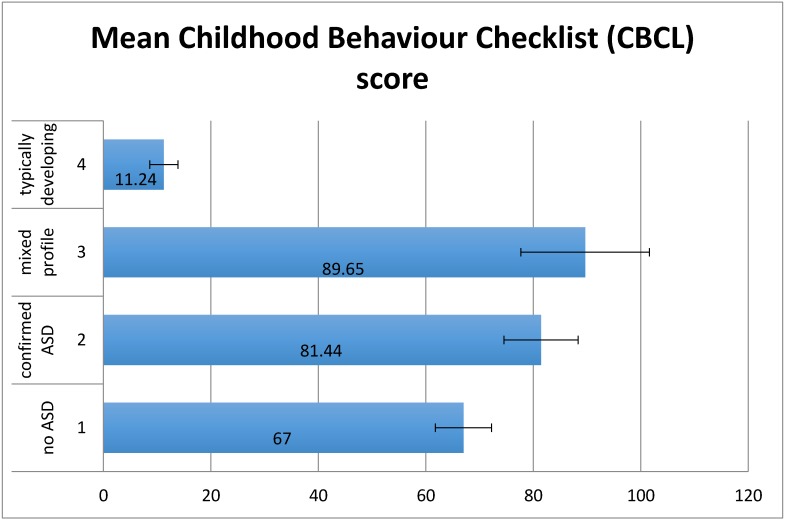
Mean CBCL scores by group. Error bars show ±1 standard error.

There was a significant effect of group on CBCL (*F*(3,88) = 19.08, *p* < 0.01) (Figure 4). There was a significant linear trend (*p* < 0.01) indicating that levels of challenging behaviour increased proportionately, with the TD group having the lowest scores followed by group 1 then group 2 then group 3. Planned contrasts showed that the TD group had significantly lower levels of challenging behaviour than groups 1–3 (*t*(88) = 7.36, *p* < 0.01), group 1 had significantly lower levels of challenging behaviour than groups 2 and 3 (*t*(88) = 2.54, *p* < 0.05), but there was no significant difference between groups 2 and 3 (*t*(88) = 0.72, *p* = 0.47). There were no significant differences between mean IQs of groups 1 to 3, although there was a significant difference (*p* < 0.01) between the mean IQ of the control group and the other 3 groups. 

## 3. Discussion

Stresses related to parenting a child with ASD [23] have been well documented. Whilst diagnosis is often a difficult and emotional time for families, receiving a diagnosis is also recognised as commonly providing a sense of relief in helping to explain a young person’s difficulties, and allows families to find and increase access to appropriate supports [24]. A main focus of this study was to examine differences between the experiences of mothers and families of young people with ASD diagnosis and a group with undiagnosed features of ASD (scoring above the SCQ cut-off for ASD but having no diagnosis). Differences between the two groups might explain why some young people receive a diagnosis and others do not, and also reveal whether the mere presence of a diagnosis might beneficially impact on maternal stress and family quality of life. 

In terms of quality of life and stress, results suggest that the presence of a young person with additional needs within the family has a detrimental effect on these and that when the young person also has an ASD (or a level of social communication difficulties commensurate with ASD but without a diagnosis) the effect is more marked. In this study, mothers in the group with high SCQ scores but without an ASD diagnosis had the lowest levels of family quality of life. Although this did not significantly differ from the group with an ASD diagnosis, it suggests that quality of life for those families for whom there is no explanation for the young person’s difficulties may be even more adversely affected. The clinical impact of this pattern may be significant despite the absence of statistical effects in this small-sample study. Whilst recognising the limitations of an association study such as this, we believe that the findings are informative to our understanding of how ASD diagnostic status may relate to family quality of life.

### What Factors Explain Group Differences?

The two groups both scoring above SCQ cut-off had the highest levels of challenging behaviour and this was also demonstrated by significant positive correlations between challenging behaviour and severity of ASD as measured by SCQ score. Higher levels of challenging behaviour were also associated with higher levels of maternal stress and lower quality of life, reinforcing previous similar findings [23,25]. 

However the two high-SCQ groups did not differ from each other in terms of severity of ASD or level of challenging behaviour. Furthermore, relations between ASD severity, stress and quality of life remained significant when controlling for challenging behaviour. This suggests that although levels of challenging behaviour have a negative influence on both stress and quality of life, the effect does not fully account for group differences. In turn, we conclude that tentative evidence of poorer quality of life and raised stress in mothers of children with signs of ASD, but no diagnosis, cannot be explained by the presence of challenging behaviours. However, what we are not able to determine is whether challenging behaviours which are intense, but of low frequency, may be having an effect on stress and quality of life. Further, it seems that challenging behaviour as a whole is not the factor that alerts professionals to a possible diagnosis of ASD. 

Young people with high SCQ scores but without an ASD diagnosis had, on average, lower IQs in this study than young people with an ASD diagnosis, which could explain the high levels of stress and poor quality of life reported by their mothers. However, this seems unlikely, as low mean IQ was also present in the group with additional needs and low SCQ scores. The IQ difference between the group with an ASD diagnosis and the group without, whilst not statistically significant, may still have been sufficiently clinically significant so as to overshadow the presence of ASD symptoms and thus reduce the likelihood of a diagnosis being made, the phenomenon of ‘diagnostic overshadowing’, being well-described in the intellectual disability literature [26,27].

Mothers of young people with additional needs experience lower quality of life and higher stress than mothers of typically developing young people. These adverse effects are stronger in the presence of ASD diagnosis, and strongest when there are signs of ASD but no diagnosis. Factors such as level of additional support, IQ and challenging behaviour cannot fully explain these relationships. An interesting topic for further investigation is whether receipt of a diagnosis itself can positively influence quality of life and levels of maternal stress.

## 4. Materials and Methods 

### 4.1. Participants and Procedure

We recruited 93 mothers of young people aged 13–22 from a much larger study of the mental health of young people within the educational system in Scotland who were considered by their teachers to be performing at an approximate IQ level of between 50 and 80 [28]. The mothers identified for this study were contacted initially by telephone and, if interested, questionnaires were sent with a letter of invitation, a consent form and information sheet. The study protocol (FSQ/05/01), information and consent sheets were approved by the UK Multi-Centre Research Ethics Committee. 

### 4.2. Measures

#### 4.2.1. Background Questionnaire

Demographic information requested included respondents’ gender, year of birth and relation to the young person. We also enquired about the medical history and type of school attended by the young person. Respondents were also asked how many contacts there had been with a range of support agencies over the previous four weeks and were asked to rate the usefulness of that contact. 

#### 4.2.2. Child Behaviour Checklist (CBCL) 

The Child Behaviour Checklist [29] is a parent/carer-completed scale and reports the activities, behaviours and functioning of the young person as previously described in more detail in Paul et al. [30]. The caregiver scores each item 0 if it is “not true”, 1 if it “sometimes or somewhat true” and 2 if it is “very true or often true” of the child. When the checklist is scored, the measure provides an overall score of behaviour in relation to population norms, as well as scores for eight syndrome scales: Withdrawn, somatic complaints, anxious/depressed, social problems, thought problems, attention problems, delinquent behaviour, and aggressive behaviour. We used the 1991 version of the scale to allow future comparison with a large dataset that collected using this scale in the Edinburgh High Risk Study [31]. The CBCL has been shown to have good reliability and validity when used in research with children and adolescents with mild intellectual disability [32,33].

#### 4.2.3. Social Communication Questionnaire (SCQ) 

The SCQ [20] is an ASD screening tool derived from the Autism Diagnostic Interview-Revised (ADI-R [34]). A 40-item parent questionnaire, the SCQ is quick and easy to use and has been well validated, showing good discriminative validity with respect to the separation of Pervasive Developmental Disorder (PDD) from non-PDD diagnoses at all IQ levels. Scores are given in three ranges: Non-PDD, PDD and autism. The cut-off score of 15 between PDD and non-PDD was most effective, with weaker discrimination between PDD and autism with best differentiation at a cut-off score of 22. In this study the cut-off of 15 was used to distinguish those with a positive screening score for ASD from those with a negative score. 

#### 4.2.4. Quality of Life; WHOQOL-BREF 

The WHOQOL-BREF [35] is a brief questionnaire assessing individual quality of life. It is a shortened version of the WHOQOL-100 assessment [36]. This measure was included as it is a widely used and well-validated standardised measure which has been tested in many settings and been shown to have good psychometric properties [35,37]. The WHOQOL-BREF generates a total score as well as sub-scores in four domains: Physical health; psychological well-being; social relations; environment. 

#### 4.2.5. Family Quality of Life Survey (FQoLS) 

The Family Quality of Life Survey [38] is a self-report questionnaire, which includes a sub-scale specifically relating to disability issues. Respondents are asked to rate, on a Likert scale, the importance of 25 aspects of family life, and are then asked to rate their satisfaction with that aspect for their own family. In the analysis the overall satisfaction score is weighted by the importance given to each of the aspects involved. The survey is reported to have relatively good internal reliability (domain scores of α = 0.82–0.90) [39].

Some adaptations were made to the original format of the FQoLS with permission from the authors. Firstly, the term ‘disability’ was replaced with ‘additional learning needs’ to fit with the terminology used in the earlier stages of the study. Secondly, as the final four items on the scale relate specifically to families of a young person with additional needs these were removed from the questionnaires completed by the typically developing group. Scores for this “disability issues” subscale were compared for the three additional needs participant groups, but in comparisons of all four participant groups, total scores without the final four items were used. 

#### 4.2.6. Family Stress and Coping Interview (FSCI) 

The Family Stress and Coping Interview [40] is a 23-item self-report questionnaire and was used to measure maternal stress. The respondent is asked to rate on a scale of 0–3 the stress currently being experienced in relation to each item. The FSCI is designed for use in families of children or young people with developmental disabilities and as such the content was not suitable for use with the families of typically developing young people; it was therefore used only with groups 1–3. The Interview has previously been reported to have high internal consistency (α = 0.89) and to discriminate between individuals with different levels of maladaptive behaviour (*F* = 3.70, *p* < 0.05) [40]. As with the FQoLS, this measure was adapted by a change of the term ‘developmental disability’ to ‘additional learning needs’, again to make this questionnaire compatible with terminology used elsewhere in the study.

### 4.3. Data Analysis 

Data were analysed using the Statistical Package for Social Sciences version 14.0 for Windows (SPSS Inc., Chicago, IL, USA). Descriptive statistics were used to investigate group characteristics and group differences, including possible confounding factors. Correlations amongst the main variables in the study were analysed using Pearson’s correlation coefficient, with partial correlations carried out to control for the effect of one variable while examining the relationship between two other variables. One-way analyses of variance (ANOVAs) with planned contrasts were used to test for the hypothesised pattern of group scoring for each variable. In the case of non-parametric data, the Kruskal-Wallis test was used to investigate group differences and Spearman’s correlation coefficient was used to examine correlations.

## Figures and Tables

**Table 1:** Demographic data by group.

		1	2	3	4
		Additional Needs			Typically Developing
		−ve SCQ −ve ASD	+ve SCQ +ve ASD	+ve SCQ −ve ASD	
		*n* = 41	*n* = 18	*n* = 17	*n* = 17
Age *	mean	15.60	16.35	16.06	15.85
	SD	1.9	2.34	1.40	1.7
Gender	no. (%) male	23 (56%)	17 (95%)	12 (71%)	8 (47%)
IQ	Mean	72.78	84.06	66.50	111.82
	SD	16.86	22.08	9.35	16.88
School	Mainstream	20 (49%)	7 (39%)	3 (18%)	15 (88%)
	Special	17 (41%)	9 (50%)	11 (65%)	0
	FE College	4 (10%)	2 (11%)	3 (18%)	2 (12%)
SCQ	mean	7.53	22.89	20.06	0.59
	SD	3.87	5.83	3.38	0.79
CBCL	Mean	67.00	81.44	89.65	11.24
	SD	33.43	29.29	49.27	10.85

* There were no significant differences in mean age between the 4 groups. ASD: Autism Spectrum Disorder; CBCL: Childhood Behaviour Checklist; IQ: Intelligence Quotient; FE: Further Education; SCQ: Social Circumstances Questionnaire; SD: Standard Deviation; −ve: Negative; +ve: Positive.

**Table 2:** Correlations between study variables.

	SCQ	CBCL	WHOQOL	FQoLS	FSCI	IQ
CBCL	*r*(76) = 0.385 *					
WHOQoL	*r*(75) = −0.349 *	*r*(75) = −0.406 *				
FQoLS	*r*(73) = −0.378 *	*r*(73) = −0.250 *	*r*(72) = 0.587 *			
FSCI	*r*(74) = 0.474 *	*r*(74) = 0.363 *	*r*(74) = −0.564 *	*r*(71) = −0.464 *		
IQ	*r*(65) = −0.147	*r*(65) = −0.125	*r*(65) = 0.028	*r*(63) = −0.071	*r*(64) = −0.057	
Age	*r*(76) = 0.155	*r*(76) = −0.034	*r*(75) = −0.065	*r*(73) = −0.204	*r*(74) = 0.157	*r*(65) = −0.400

* Significant at *p* < 0.001.

**Table 3:** Partial correlations controlling for challenging behaviour levels.

Control Variable		SCQ
CBCL	FQoLS	−0.319 *
	FSCI	0.369 *

* Significant at *p* < 0.001.

## References

[1] American Psychiatric Association (2013). Diagnostic and statistical manual of mental disorders (5th ed.).

[2] Elsabbagh M., Divan G., Koh Y.J., Kim Y.S., Kauchali S., Marcin C., Montiel-Nava C., Patel V., Paula C.S., Wang C. (2012). Global prevalence of autism and other pervasive developmental disorders. Autism Res..

[3] Simonoff E., Pickles A., Charman T., Chandler S., Loucas T., Baird G. (2008). Psychiatric disorders in children with autism spectrum disorders: Prevalence, comorbidity, and associated factors in a population-derived sample. J. Am. Acad. Child Adolesc. Psychiatry.

[4] McClintock K., Hall S., Oliver C. (2003). Risk markers associated with challenging behaviours in people with intellectual disabilities: A meta-analytic study. J. Intellect Disabil. Res..

[5] Matson J.L., Rivet T.T. (2008). Characteristics of challenging behaviours in adults with autistic disorder, PDD-NOS, and intellectual disability. J. Intellect Dev. Disabil..

[6] Stores R., Stores G., Fellows B., Buckley S. (1998). Daytime behaviour problems and maternal stress in children with down’s syndrome, their siblings, and non- intellectually disabled and other intellectually disabled peers. J. Intellect Disabil. Res..

[7] Zablotsky B., Anderson C., Law P. (2013). The association between child autism symptomatology, maternal quality of life, and risk for depression. J. Autism Dev. Disord..

[8] Fombonne E., Simmons H., Ford T., Meltzer H., Goodman R. (2001). Prevalence of pervasive developmental disorders in the british nationwide survey of child mental health. J. Am. Acad. Child Adolesc. Psychiatry.

[9] Wolf L.C., Noh S., Fisman S.N., Speechley M. (1989). Psychological effects of parenting stress on parents of autistic children. J. Autism Dev. Disord..

[10] Dillenburger K., Jordan J.A., McKerr L., Keenan M. (2015). The millennium child with autism: Early childhood trajectories for health, education and economic wellbeing. Dev. Neurorehabil..

[11] Blacher J., McIntyre L.L. (2006). Syndrome specificity and behavioural disorders in young adults with intellectual disability: Cultural differences in family impact. J. Intellect Disabil. Res..

[12] Douma J.C., Dekker M.C., Koot H.M. (2006). Supporting parents of youths with intellectual disabilities and psychopathology. J. Intellect Disabil. Res..

[13] Hassall R., Rose J., McDonald J. (2005). Parenting stress in mothers of children with an intellectual disability: The effects of parental cognitions in relation to child characteristics and family support. J. Intellect Disabil. Res..

[14] (2006). Autistic Spectrum Disorders Needs Assessment Report (2001): Scottish Executive Report on Implementation and Next Steps.

[15] McKenzie K., Forsyth K., O’Hare A., McClure I., Rutherford M., Murray A., Irvine L. (2015). Factors influencing waiting times for diagnosis of autism spectrum disorder in children and adults. Res. Dev. Disabil..

[16] Keenan M., Dillenburger K., Doherty A., Byrne T., Gallagher S. (2010). The experiences of parents during diagnosis and forward planning for children with autism spectrum disorder. J. Appl. Res. Intellect Disabil..

[17] Crane L., Chester J.W., Goddard L., Henry L.A., Hill E. (2016). Experiences of autism diagnosis: A survey of over 1000 parents in the united kingdom. Autism.

[18] Howlin P., Moore A. (1997). Diagnosis in autism: A survey of over 1200 patients in the uk. Autism.

[19] Howlin P., Asgharian A. (1999). The diagnosis of autism and asperger syndrome: Findings from a survey of 770 families. Dev. Med. Child Neurol..

[20] Berument S.R.M., Lord C., Pickles A., Bailey A. (1999). Autism screening questionnaire: Diagnostic validity. Br. J. Psychiatry.

[21] Wechsler D. (1999). Wechsler adult intelligence scale.

[22] Wechsler D. (1992). Wechsler intelligence scale for children.

[23] Lecavalier L., Leone S., Wiltz J. (2006). The impact of behaviour problems on caregiver stress in young people with autism spectrum disorders. J. Intellect Disabil. Res..

[24] Chamak B., Bonniau B., Oudaya L., Ehrenberg A. (2011). The autism diagnostic experiences of french parents. Autism.

[25] Baker B.L., Blacher J., Crnic K.A., Edelbrock C. (2002). Behavior problems and parenting stress in families of three-year-old children with and without developmental delays. Am. J. Ment. Retard..

[26] Reilly C., Senior J., Murtagh L. (2015). ASD, ADHD, mental health conditions and psychopharmacology in neurogenetic syndromes: Parent survey. J. Intellect Disabil. Res..

[27] Rush K.S., Bowman L.G., Eidman S.L., Toole L.M., Mortenson B.P. (2004). Assessing psychopathology in individuals with developmental disabilities. Behav. Modif..

[28] Johnstone E.C., Owens D., Hoare P., Gaur S., Spencer M.D., Stanfield A., Moffat V., Harris J.M., Brearley N., Miller P.M. (2007). Schizotypal cognitions as a predictor of psychopathology in adolescents with mild intellectual impairment. Br. J. Psychiatry.

[29] Achenbach T.M. (1991). Manual for the child behavior checklist/4–18 and 1991 profile.

[30] Paul A.R., McKechanie A.G., Johnstone E.C., Owens D.G., Stanfield A.C. (2015). Brief report: The association of autistic traits and behavioural patterns in adolescents receiving special educational assistance. J. Autism Dev. Disord..

[31] Johnstone E.C., Ebmeier K.P., Miller P., Owens D.G., Lawrie S.M. (2005). Predicting schizophrenia: Findings from the Edinburgh high-risk study. Br. J. Psychiatry.

[32] Schachter D.C., Pless I.B., Bruck M. (1991). The prevalence and correlates of behaviour problems in learning disabled children. Can. J. Psychiatry.

[33] Crijnen A.A., Achenbach T.M., Verhulst F.C. (1999). Problems reported by parents of children in multiple cultures: The child behavior checklist syndrome constructs. Am. J. Psychiatry.

[34] Lord C., Rutter M., Le Couteur A. (1994). Autism diagnostic interview-revised: A revised version of a diagnostic interview for caregivers of individuals with possible pervasive developmental disorders. J. Autism Dev. Disord..

[35] WHOQOL Group (1998). Development of the World Health Organization WHOQOL-BREF quality of life assessment. Psychol. Med..

[36] WHOQOL Group (1995). The World Health Organization Quality of Life assessment (WHOQOL): Position paper from the World Health Organization. Soc. Sci. Med..

[37] Skevington S.M., Lofty M., O’Connell K.A. (2004). The World Health Organization’s WHOQOL-BREF quality of life assessment: Psychometric properties and results of the international field trial. A report from the WHOQOL group. Qual. Life Res..

[38] Poston D., Turnbull A., Park J., Mannan H., Marquis J., Wang M. (2003). Family quality of life: A qualitative inquiry. Ment. Retard.

[39] Park J., Hoffman L., Marquis J., Turnbull A.P., Poston D., Mannan H., Wang M., Nelson L.L. (2003). Toward assessing family outcomes of service delivery: Validation of a family quality of life survey. J. Intellect Disabil. Res..

[40] Nachshen J.S., Woodford L., Minnes P. (2003). The family stress and coping interview for families of individuals with developemntal disabilities: A lifespan perspective on family adjustment. J. Intellect Disabil. Res..

